# Generation of different sizes and classes of small RNAs in barley is locus, chromosome and/or cultivar-dependent

**DOI:** 10.1186/s12864-016-3023-5

**Published:** 2016-09-15

**Authors:** Michael Hackenberg, Antonio Rueda, Perry Gustafson, Peter Langridge, Bu-Jun Shi

**Affiliations:** 1 Computational Genomics and Bioinformatics Group, Genetics Department, University of Granada, 18071 Granada, Spain; 2 Genomics and Bioinformatics Platform of Andalusia (GBPA), Edificio INSUR, Calle Albert Einstein, 41092 Seville, Spain; 3 USDA-ARS, 206 Curtis Hall, University of Missouri, Columbia, MO 65211-7020 USA; 4 Australian Centre for Plant Functional Genomics, The University of Adelaide, Adelaide, South Australia 5005 Australia

**Keywords:** Small RNA expression, Origin, Chromosome location, Conservation, Barley cultivar

## Abstract

**Background:**

Various small RNA (sRNA) sizes and varieties have been identified, but their relationship as well as relationship with their origins and allocations have not been well understood or investigated.

**Results:**

By comparing sRNAs generated from two barley cultivars, Golden Promise (GP) and Pallas, we identified that the generation of different sizes and types of sRNAs in barley was locus-, chromosome- and/or cultivar-dependent. 20-nt sRNAs mainly comprising miRNAs and chloroplast-derived sRNAs were significantly over-expressed in Pallas vs. GP on chromosomes 3H and 6H. MiRNAs-enriched 21-nt sRNAs were significantly over-expressed in Pallas vs. GP only on chromosome 4H. On chromosome 5H this size of sRNAs was significantly under-expressed in Pallas, so were 22-nt sRNAs mainly comprising miRNAs and repeat-derived sRNAs. 24-nt sRNAs mostly derived from repeats were evenly distributed in all chromosomes and expressed similarly between GP and Pallas. Unlike other sizes of sRNAs, 24-nt sRNAs were little conserved in other plant species. Abundant sRNAs were mostly generated from 3’ terminal regions of chromosome 1H and 5’ terminal regions of chromosome 5H. Over-expressed miRNAs in GP vs. Pallas primarily function in stress responses and iron-binding.

**Conclusions:**

Our study indicates that 23−24-nt sRNAs may be linked to repressive chromatin modifications and function in genome stability while 20−21-nt sRNAs may be important for the cultivar specificity. This study provides a novel insight into the mechanism of sRNA expression and function in barley.

**Electronic supplementary material:**

The online version of this article (doi:10.1186/s12864-016-3023-5) contains supplementary material, which is available to authorized users.

## Background

Small RNAs (sRNAs) carry out important functions in plants. MicroRNAs (miRNAs) and short interfering RNAs (siRNAs) are two major classes of small non-coding regulatory RNAs, ranging in size from 20 to 24 nucleotides (nt). However, miRNAs are single-stranded RNAs derived from hairpin precursors (pre-miRNAs), which are processed from miRNA primary transcripts (pri-miRNAs) transcribed from genomic DNA, while siRNAs are double-stranded RNAs derived from transposable elements (TEs), tandem repeats, convergent mRNA transcripts, natural sense-antisense pairs, duplexes involving pseudogene-derived antisense transcripts and the sense mRNAs from their cognate genes, and hairpin RNAs. siRNAs can also be produced from exogenous sources like RNA viruses and transgenes. In addition, one miRNA locus only produces one miRNA duplex while one siRNA locus can generate many siRNA duplexes [1]. Furthermore, miRNAs can be conserved between plant and animal [2] while siRNAs are rarely conserved in related organisms [3]. These differences can be used to distinguish between miRNAs and siRNAs [4]. Despite of the difference, the biogenesis and function of miRNAs and siRNAs are similar. Both miRNAs and siRNAs depend upon Dicer enzymes for generation and the Argonaute (AGO)-containing RNA-induced silencing complexes (RISCs) for function. Plant miRNAs are mostly associated with AGO1 [5]. In addition both miRNAs and siRNAs recognise mRNA targets based on their perfect or nearly perfect antisense complementarity and cleave the mRNA targets near the middle of the complementarity regions [6].

Upon the nature, endogenous siRNAs can be named as heterochromatic siRNAs, secondary siRNAs and natural antisense transcript siRNAs (nat-siRNAs). 23−24-nt (especially 24-nt) heterochromatic siRNAs are derived from intergenic and/or repetitive genomic regions and are a major class of endogenous siRNAs [7]. They are generated by RNA-dependent RNA polymerase 2 (RDR2) and Dicer-like 3 (DCL3), which depends on the changes of transposon position and copy number during evolution [7]. 21-nt secondary siRNAs, including phased siRNAs (phasiRNAs) or trans-acting siRNAs (ta-siRNAs) and nat-siRNAs that act in cis or in trans, are derived from dsRNA precursors and triggered by other sRNAs together with DCL4 and RDR6 [7]. In the absence of DCL4, DCL2 can step in to produce 22-nt siRNAs [7]. Recent studies showed that secondary ta-siRNAs and cis-nat-siRNAs produced from overlapping protein-coding genes can be triggered by 22-nt miRNAs [8]. Either 21-nt or 22-nt siRNAs are associated with AGO1 and guide the cleavage of mRNA targets. By contrast, 23−24-nt siRNAs are associated with AGO4 or AGO6 and promote DNA methylation in asymmetric CHH sites and H3K9 histone methylation at the target DNA loci to silence transposon activity for maintaining genome integrity [9].

Barley (*Hordeum vulgare*) is an important cereal crop and good genetic model among Triticeae species. Many varieties exist and all of them contain seven chromosomes (1H–7H). In this study, we compared sRNA profiles genome-widely between two distinct barley cultivars, *H. vulgare* L. cv. Golden Promise (GP) and *H. vulgare* L. cv. Pallas. We found many cultivar-specific or significantly differentially expressed sRNAs between the cultivars. Remarkably, we found that the generation of different sizes or types of sRNAs was locus, chromosome and/or cultivar-dependent, and only 20–22-nt, but not 24-nt, sRNAs were conserved in other plant species. To our knowledge, this is the first genome-wide identification of the relationship between sRNA generation and allocation in plants, and the conservation of sRNAs among different plant species.

## Methods

### Barley growth under a controlled environmental condition

Two barley cultivars, GP and Pallas, available at the Australian Centre for Plant Functional Genomics were selected for comparison in this study. GP is a gamma-ray induced semi-dwarf mutant of the cultivar ‘Maythorpe’, has been the subject of many genetic studies including pedigree analysis and genome scanning, and is an extremely important barley cultivar [10]. Pallas is a high-yielding X-ray mutant of the cultivar “Bonus” and was among the first cereal mutants released into practice. This mutant cultivar has been widely used for plant breeding [11]. Both GP and Pallas were grown under two conditions: well water and water below −5 bars. Each cultivar/treatment had 6 replicates. Imaging and watering were taken at the same time every 2 days from 30 to 70 days after sowing using the Lenmatec platform. Soil water potential was estimated from leaf water potential of GP plants. The average projected area viewed from two sides and top side of the plants was used to create growth plots and to calculate final leaf area, growth rate and time of inflexion point at which the growth rate started to decrease, which was considered as a transition from the vegetative stage to the reproductive stage.

### sRNA isolation and sequencing

sRNAs were isolated using the Purelink miRNA isolation kit (Invitrogen, Carlsbad, CA, USA) from leaf material pooled from 3 individual plants of each of GP and Pallas cultivars after three weeks of germination and growth under well water conditions. The same concentration of sRNAs from each sample was used for library preparation and sRNA sequencing was performed in the same flow cell in the Illumina platform. These measures minimised artificial differences.

### Bioinformatics analysis, prediction and GO analysis of miRNAs’ targets and genome distribution analysis

Bioinformatics analysis was performed using sRNAbench [12], a new tool based on miRanalyzer [13]. Briefly, the pre-processing of the reads consisted in the following steps: i) the 5’ adapter was trimmed forcing the detection of at least 10 nt of the adapter sequence within the read allowing 1 mismatch; ii) untrimmed reads, short reads (<15 nt) and reads with ambiguous nucleotides are filtered out; and iii) the remaining reads are collapsed into unique reads assigning to each unique read count (i.e. the number of times the read was obtained in the sequencing experiment). The adapter-cleaned reads were then mapped to the barley genome by means of the Bowtie aligner [14]. Prediction and Gene Ontology (GO) analysis of miRNAs’ targets were performed as previously described [15–18]. The genome distribution was analysed using the Bowtie alignment files. Details of these analyses are described in Additional file 1.

## Results

### Growth rate of barley cultivars GP and Pallas

Under well-water condition both GP and Pallas cultivars showed no difference in developmental time, but Pallas grew at a higher rate than GP (Additional file 2: Figure S1). Under drought condition both cultivars had neither difference in developmental time nor difference in growth rate (Additional file 2: Figure S1).

### sRNA generation from GP and Pallas

Total 6482301 and 4509869 clean reads were generated from GP and Pallas, respectively, using the Illumina’s sequencing technology (Additional file 3: Table S1). Size distribution revealed a typical plant sRNA pattern with two local maxima at 20/21 nt and 24 nt. While the 20/21 nt peak had the highest number of reads, the 24 nt peak was comprised by the highest number of low-copy unique reads, in both GP and Pallas (Additional file 2: Figure S2A, B).

83.69 % of the reads (or 70.86 % unique reads) in GP and 80.41 % (or 68.12 % unique reads) in Pallas were perfectly mapped to the genomes of barley cultivars Morex, Barke and Bowman (Additional file 4: Table S2). The mapped reads from both cultivars showed a similar size distribution after the adapter was removed and no appreciable contamination or differences in sequencing quality existed (Additional file 2: Figure S2C, D).

**Fig. 1:**
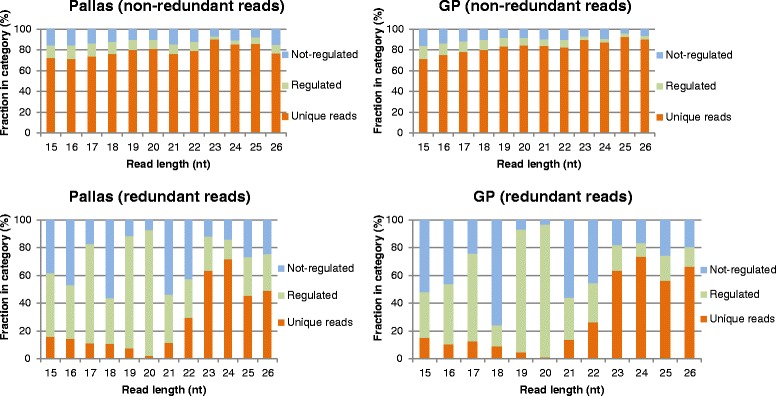
The relative frequencies of cultivar specific, regulated and non-regulated reads within each read length for unique (non-redundant) reads and read count (redundant reads). Barley cultivars are indicated

Cross comparison yielded 618839 GP-specific unique reads with a total read count of 748936 (16.9 %) and 505572 Pallas-specific unique reads with a total read count of 613781 (13.8 %) (Additional file 5: Table S3). Only 106706 unique reads were shared between the two cultivars with a total read count of 4675854 (83.1 %) in GP and 3012754 (86.2 %) in Pallas (Additional file 5: Table S3), indicating that most unique reads are not shared and most sequenced reads are common between both cultivars. However, drastic differences were observed for the degree of shared reads as a function of read length (Fig. 1). Long reads were found to contain a higher percentage of cultivar-specific unique reads than short reads in both cultivars. This gap was even larger when the total read count was considered. The greatest difference occurred between 20-nt and 24-nt reads (only 2 % of 20-nt reads were cultivar-specific while 70 % of 24-nt reads were cultivar-specific) in both cultivars. The 24-nt reads, which were considered as the putative heterochromatic siRNAs, were the most cultivar-specific sRNA population. However, the shared 24-nt reads were the least (around 14 %), while the shared 20-nt reads were the most (over 90 %), differentially expressed RNA populations as defined by log(differential expression) = (RPM(GP) + 0.1) / (RPM (Pallas) + 0.1), where RPM represents reads per million calculated by dividing the read count by the total reads in the dataset and then multiplying by 10^6^. The added 0.1 is to avoid division by 0.

Other read lengths like 15, 16 and 17 nt or longer reads of 25 and 26 nt also existed in the datasets in minor frequencies. However, they unlikely resulted from a pure random degradation pathway. Instead, part of them may be produced via a processing pathway because of their different percentages, for example, less than 20 % for short reads and over 50 % on average for longer reads. Whether these sRNAs have biological functions is unknown. Notably, genomic sequences for some cultivar-specific sRNAs existed in both cultivars, thereby raising the possibility that trans-acting factors, which might be different between the cultivars, may be involved in the transcription of the cultivar-specific sRNAs.

### Expression of sRNA types within and between the cultivars

The genome-mapped reads were further classified using sRNAbench [12] and the barley genome annotation (http://plants.ensembl.org/Hordeum_vulgare/Info/Index) as well as other specific databases like the Rfam database [19], miRBase [20], the TIGR repeat database [21], the TREP repeat database [22], RepBase [23] and the genomic tRNA database [24]. 16.80 % and 30.21 % of the read count from GP and Pallas, respectively, were miRNA sequences, 13.21 % and 17.43 % of the read count from GP and Pallas, respectively, were repetitive sequences, 5.58 % and 3.56 % of the read count from GP and Pallas, respectively, were tRNA sequences, and 1.67 % and 2.21 % of the read count from GP and Pallas, respectively, were rRNA sequences (Additional file 6: Table S4). In addition, 8.24 % of the read count from GP and 9.49 % of the read count from Pallas were mapped to biological regions where no specific details were given or defined. 35.78 % of the read count from GP and 15.54 % of the read count from Pallas were mapped to the chloroplast genome (note: the nuclear genome also contains chloroplast genome sequences). A chloroplast-derived sRNA that mapped tRNA-His(GUG) gene was the most abundant of all the sRNAs. However, sRNAs mapped to gene regions, small nuclear RNAs (snRNAs) or small nucleolar RNAs (snoRNAs) were limited. We defined the sRNAs that were not related to miRNAs and structural RNAs (ribosomal RNAs (rRNAs), transfer RNAs (tRNAs), snRNAs etc.) as siRNAs. siRNAs were more in GP than in Pallas. Some siRNAs were derived from coding sequence (CDS), the 5’ and 3’ untranslated regions (UTRs) and introns while others were from antisense strands of these regions. For better comparison, read count of each sRNA from each cultivar was normalized as RPM. This normalization led the miRNA-mapped reads in Pallas and the chloroplast genome-mapped reads in GP to the highest (Additional file 2: Figure S3A). In terms of unique reads, repeats-derived, biological regions-derived and unassigned reads were dominant but not significantly differentially expressed between the cultivars (Additional file 2: Figure S3B). Overall, the reads mapped to tRNAs, gene regions, chloroplast genome, snRNAs and snoRNAs were less in Pallas than in GP, while the reads mapped to miRNAs, rRNAs, repeats, biological regions and unassigned were more in Pallas than in GP (Additional file 2: Figure S3C).

### Association of sRNA sizes with sRNA types within and between the cultivars

**Fig. 2:**
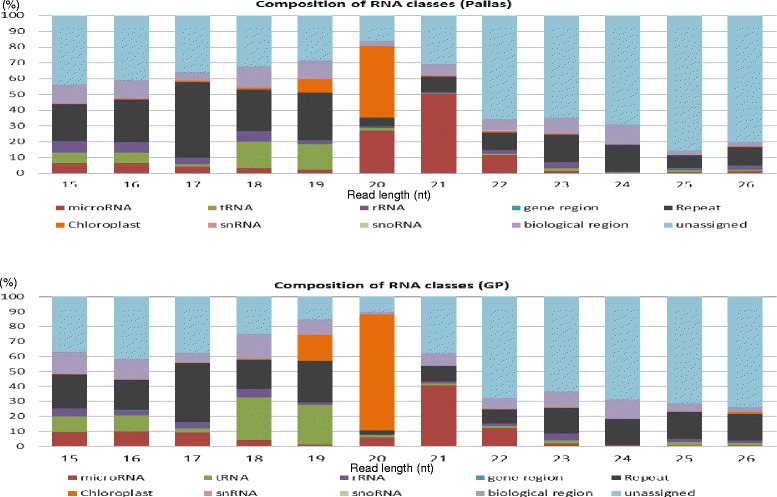
The percentages of different types of sRNAs in each size of the genome-mapped reads in Pallas and in Golden Promise (GP)

21-nt sRNAs contained 40−50 % miRNA sequences, whereas 24-nt sRNAs almost contained no miRNA sequences, in each cultivar (Fig. 2). On the other hand, the 24-nt sRNAs contained 17 % repeat sequences, whereas the highly abundant 21-nt sRNAs only contained 10 % repeat sequences. Furthermore, almost all the chloroplast-derived sequences were contained in 19–20-nt sRNAs, which were more in GP (77.51 % of 19–20-nt sRNAs contained chloroplast-derived sequences) than in Pallas (45.40 % of 19–20-nt sRNAs contained chloroplast-derived sequences). However, the miRNA sequences-contained 20-nt sRNAs were more in Pallas (27.03 % of 20-nt sRNAs contained miRNA sequences) than in GP (6.09 % of 20-nt sRNAs contained miRNA sequences), suggesting that the numbers or processing activities of miRNA genes or miRNA stabilities may be different between the two cultivars. Some sRNAs were derived from both sense and antisense strands of intergenic regions, CDS, 3’ and 5’ UTRs, introns, and up- and down-stream gene regions, but more from the sense strands than from the antisense strands. 20-nt sRNAs in GP and 19-nt sRNAs in Pallas were dominantly mapped to antisense strands, suggesting that they are controlled by different mechanisms in each cultivar. All size sRNAs that were mapped to elements in each cultivar are shown in Additional file 2: Figure S4.

### Genome-wide distribution of different sizes and types of sRNAs within and between the cultivars

**Fig. 3:**
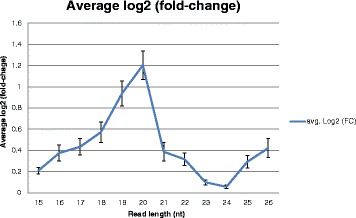
Average log2 of the fold change between the relative frequencies of Pallas and GP chromosomes. Reads with length 24 nt are by far those with the smallest fold-changes. The error bars show the standard error.

To analyse the intra and inter-chromosome distribution of the sRNAs between the two cultivars, we mapped (without mismatches) all reads to two sets of the barley genome sequences (http://plants.ensembl.org/Hordeum_vulgare/Info/Index) (Additional file 7: Table S5). One set labelled as 1H to 7HS/L is the whole genome shotgun (WGS) contigs in context of the coordinates provided by the fingerprint contigs. Another set labelled 1–7 is the concatenated sequences of all anchored WGS contigs and bacterial artificial chromosome (BAC) sequences. We first determined the relative frequencies for all chromosomes as a function of the read length. As shown in Fig. 3, 24-nt reads had the most equal relative frequencies on the different chromosomes between GP and Pallas, whereas 20-nt reads was contrary. The mean log2 of the fold-changes is only 0.05 for 24-nt reads, while it is 1.2 for 20-nt reads.

**Fig. 4:**
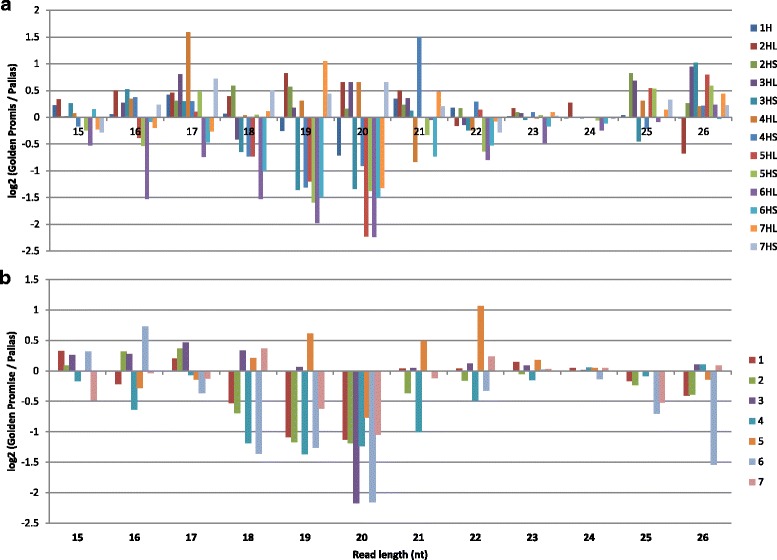
Allocation and differential expression of different sizes of sRNAs in the barley genome between GP and Pallas. **a**. Allocation and differential expression of different sizes of sRNAs at the chromosomes labelled as 1H to 7HS/L from the whole genome shotgun (WGS) contigs. **b**. Allocation and differential expression of different sizes of sRNAs at the chromosomes labelled as 1–7 from the concatenated sequences as described in the text

Next, we determined distribution of different sizes and types of sRNAs on each chromosome. 70 % and 44.2 % of 20-nt sRNAs from GP and Pallas, respectively, were found to gather on chromosomes 2HL, 3HL, 4HL and 7HS (Fig. 4a). To other chromosomes, only a minor fraction of 20-nt sRNAs was aligned. On chromosome 3HS, only 0.04 % 20-nt sRNAs from GP and 0.10 % from Pallas were distributed. Although the other sizes of sRNAs were distributed in each chromosome evenly, in terms of the long and short arms, 24-nt sRNAs were distributed more in the long arms than in the short arm in every chromosome in both cultivars.

20-nt sRNAs were also over-expressed on all chromosomes, especially chromosomes 3H and 6H, in Pallas, where 19-nt sRNAs behaved differently (Fig. 4b). 21-nt sRNAs were under-expressed on chromosome 5H, but over-expressed on chromosomes 2H, 4H and 7H, in Pallas. Among all sizes of sRNAs 22-nt sRNAs were the most significantly under-expressed in Pallas especially on chromosome 5H.

**Fig. 5:**
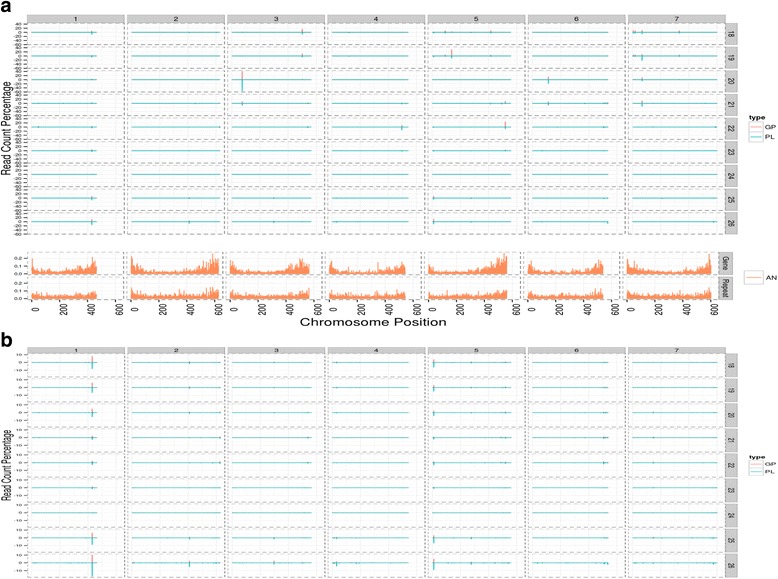
Spatial chromosomal distribution of the sequencing reads as a function of its length. The graphic shows the logarithm to the base of 2 of the relative frequencies of unique reads (*top*) and relative frequencies of read counts (*bottom*) for GP (*positive values*) and Pallas (*negative values*). Note that the relative frequencies are obtained per read length and therefore the peak heights cannot be compared among the different read lengths but only between the different chromosomes for a given read length

Abundant sRNAs were found to be generated mainly from the 3’ region of chromosome 1H and the 5’ region of chromosome 5H (Fig. 5a, b). 25−26-nt sRNAs were mostly generated from the 3’ region of chromosome 1H (Fig. 5a, b). The other regions of chromosome 5H, the 3’ region of chromosome 6H, the 5’ region of chromosome 4H and middle regions of the other chromosomes generated less abundant sRNAs (Fig. 5a, b). However, all chromosomes generated 24-nt sRNAs and furthermore evenly between the chromosomes regardless of their different sequences (Fig. 5a, b). This may be due to that most 24-nt sRNAs were derived from repeats, which were evenly distributed on every chromosome (Fig. 5a) second panel﻿ in the last graphic. We also found that all chromosomes generated gene-derived sRNAs, but not evenly (Fig. 5a first panel﻿ in the last graphic). By means of sRNAtoolbox [25] we generated the genome distribution of sRNAs as a function of read length for both cultivars. The sRNA data together with Ensembl annotations can be accessed by means of the following link: http://bioinfo5.ugr.es/srnatoolbox/barleyCultivar.

Four genomic regions (morex_contig_1658654, morex_contig_1656108, morex_contig_2556638 and morex_contig_175283) showed significant differences in sRNA distribution between the two cultivars (Additional file 8: Table S6), i.e., morex_contig_1658654 and morex_contig_1656108-derived sRNAs were much more in Pallas than in GP, whereas those derived from morex_contig_2556638 and morex_contig_175283 were the other way around. These regions could be used as an index to distinguish between the two cultivars.

### Conservation of barley sRNAs in other plant species

**Fig. 6:**
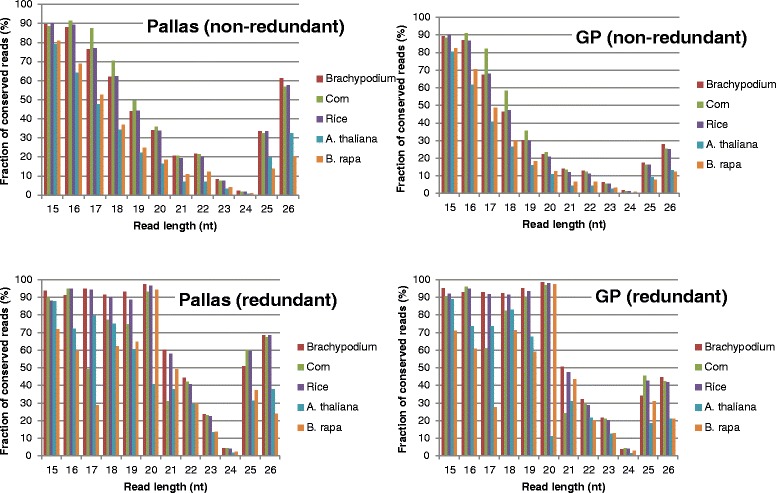
The fraction of mapped reads as a function of read length in 5 indicated plant genomes. The fraction is an estimate of the degree of conserved reads. Redundant and non-redundant reads from each of GP and Pallas are labelled

Although a massive number of sRNAs have been sequenced from various species in the past, the conservation of sRNAs between species had not been investigated. To explore this information and find possible sequence-depending functions of the different sRNA populations, we mapped all reads from GP and Pallas to the genomes of 5 other plant species, *Brachypodium*, rice, maize, *Brassica rapa* and *Arabidopsis* (Fig. 6). *Barchypodium* is the phylogenetically closest to barley and together with rice and maize are monocots, while the other two species are dicots. Surprisingly, 24-nt unique reads were the lowest mapped to the 5 genomes (only around 1 %) of all sizes of reads despite that they had the highest unique read number. 20-nt unique reads were the highest mapped to the 5 genomes of all sizes of reads. Over 90 % of the total 20-nt read count was conserved in the plant species except *Arabidopsis*. Depending on the species and the cultivar the mapping percentage of 20-nt unique reads is between 11 % (GP vs. *Arabidopsis*) and 35.9 % (Pallas vs. maize). Mapped sRNAs from Pallas were more than those from GP. As expected, mapped sRNAs to the monocot plants were higher than those to the dicot plants. 15–18-nt and 25–26-nt reads were also highly conserved in the 5 plant genomes, suggesting that the conserved reads may be generated from conserved longer RNA molecules.

### Expression and function of miRNAs within and between the cultivars

MiRNAs in both cultivars were particularly analysed. A total of 89 conserved miRNAs were identified, of which 9 were GP-specific, 6 were Pallas-specific, and 10 and 6 miRNAs were significantly up- and down-regulated (|log2| > 1), respectively, in GP (Additional file 9: Table S7). Interestingly, members in some miRNA families were differentially expressed between the two cultivars. For example, hvu-miR399e-5p, hvu-miR399c-5p and hvu-miR399c-3p (26) were only present in Pallas, while hvu-miR399d-3p [26] was only present in GP (Additional file 9: Table S7). This suggests that some miRNAs may be cultivar-dependent expressed and/or have a role in cultivar specificity. Apart from the identified conserved miRNAs, we also identified 117 novel miRNAs, of which 7 were absent, 5 were significantly up-regulated (log2 > = +1) and 35 were significantly down-regulated (log2 = < −1) in Pallas (Additional file 9: Table S7). However, these novel miRNAs were all weakly expressed and additionally, most of them are encoded by single loci and of 22 nt in size, which is consistent with previous studies [27]. For convenience and distinction, these novel miRNAs were temporally given a name starting with “X” (Additional file 9: Table S7).

Ten miRNAs (indicated in Additional file 9: Table S7) were selected for validation of their differential expression between the leaf tissues of two barley cultivars using quantitative real-time PCR (qRT-PCR). In the sRNA datasets six of these selected miRNAs were up-regulated, three were down-regulated in GP and another one was unchanged between the two cultivars. The qRT-PCR result showed that four miRNAs were up-regulated and three were down-regulated in GP, which were consistent with their read abundances in the sRNA datasets (Additional file 2: Figure S5). The remaining three miRNAs failed to generate a uniform RT-PCR product and cannot be conclusively.

To verify the function of the differentially expressed miRNAs, their targets were predicted using psRNATarget [16]. 94 targets of upregulated miRNAs in Pallas (Additional file 10: Table S8) and 516 targets of upregulated miRNAs in GP were predicted (Additional file 11: Table S9). GO analysis showed that the predicted targets encode a wide range of protein functions, including transcription factors, kinases, oxidoreductases, hydrolases, transferases, receptors and transporters (Additional file 12: Table S10). Of these functions, stress response and iron binding functionalities were the most significant in GP. Target genes with mentioned functions were 18 times (corrected *p*-value < = 1.97*10^−4^) and 47.7 times (corrected *p*-value < = 0.002) more frequent than expected by chance, respectively (Additional file 2: Figure S6). Functionalities of miRNAs in the hormone signalling pathway, RNA cellular processing and energy mobilization were also significant in GP. In contrast, neither of these functionalities was significant in Pallas (compare Additional file 2: Figure S6A and 6B). High levels of miRNA target divergence had previously been observed between *A. thaliana* and *A. lyrata* [28]. Thus, our data suggests that the genes regulated by up-regulated miRNAs in GP or in Pallas could have a potential involvement in the phenotypic differences of the two barley cultivars.

## Discussion

Genome-wide comparison of sRNAs from two distinct cultivars can gain a better understanding of the regulation of plant cellular process mediated by miRNAs and other sRNAs. In this study, we found that many sRNAs including miRNAs were differentially expressed between the barley cultivars. Whether a cultivar-associated factor is present that is used to control the sRNA expression for possibly directing cultivar specificities is interesting. Previous studies suggested that the differently expressed miRNAs might result from newly generated loci through inverted duplication events and/or duplications of transcribed, protein-coding genes [5]. This suggestion has been supported by the observation of a high proportion of species-specific or non-conserved miRNA genes in many plant species, including *Physcomitrella patens* [29], *Selaginella moellendorffii* [28], rice [30–33], *Medicago truncatula* [34, 35] and *Glycine max* [36]. However, it cannot be excluded that the differently expressed miRNAs or sRNAs may also possibly result at some very specific developmental point. Generally, differential expression of the cultivar-associated miRNAs or sRNAs is complicated. For example, the differentially expressed miR159b, which targets two GAMYB-like transcription factors, MYB33 and MYB65, in *Arabidopsis* [37], had many differentially expressed sequence and length variants (termed as isomirs). However, the region encompassing this miRNA and its upstream promoter does not display any difference in nt sequence between the two cultivars, nor in other barley cultivars such as Morex, Barke and Bowman (data not shown). This could suggest that cultivar-specific trans-acting factors might be present, involved, and responsible for the differential expression. The so-called cultivar-specific trans-acting factors could possibly interact with cis-regulatory elements such as binding sites that control the transcription or interact with the post-transcriptional machineries such as Dicers to governor the processing of the differentially expressed miRNA and its isomirs. The presence of so-called cultivar-specific trans-acting factors could partly explain the presence of the cultivar-associated miRNAs and other sRNAs in the plants.

Two major sizes (21-nt and 24-nt) of sRNAs were revealed in both cultivars. The 21-nt sRNAs were highly redundant. About 41 % of 21-nt sRNAs were cultivar-specific or differentially expressed, making the most differently expressed sRNA population. The majority of the differently expressed 21-nt sRNAs originated from chromosome 5HS, suggesting that this chromosome could be a region in response to the cultivar specificities. By contrast, the 24-nt sRNAs were least redundant. Only a small portion of 24-nt sRNAs were significantly differently expressed, despite that this size of sRNAs accounted for the most of all unique sRNAs in both cultivars. Strikingly, the 24-nt sRNAs were found to be by far those that are most even distributed among the chromosomes between the barley cultivars and be the least conserved sRNA population of all sRNA populations in other plant genomes. In another aspect, this result indicated that this size of sRNAs are endogenous. These results suggest that the 24-nt sRNAs might not act as regulators of other molecules like miRNAs, but maintain the genome integrity as previously proposed [9]. On this point, some 24-nt sRNAs would function as heterochromatic siRNAs that contribute to heterochromatin formation at repetitive DNA loci. This could be true as the annotated 24-nt sRNAs, which were randomly generated in the genome and largely derived from long terminal repeat (LTR) retrotransposons, mainly targeted centromeric regions that primarily constitute TE [38]. It is to note that most 24-nt sRNAs couldn’t be assigned to a genome annotation. Intriguingly, in plants the role of heterochromatic siRNAs could be compensated by 21-nt siRNAs that are diverse, generated by TE activity [39], can silence TEs that share the complementary sequence [40] and particularly can interact with the 24-nt siRNA transcriptional silencing pathway [41]. What is a precise functional mechanism of 24-nt and 21-nt siRNAs in silencing transposon activity at the repeat loci is unknown and needs to be defined experimentally. Curiously, the 24-nt heterochromatin siRNAs were found to be located differently within a chromosome or between the chromosomes or between the cultivars, and furthermore more in Pallas than in GP. These imply that the factors involved in the generation of heterochromatin siRNAs might be different in different chromosomes and/or different cultivars. However, the exact generation mode of heterochromatic siRNAs is not yet known.

## Conclusions

Our data show that the generation of different sizes and types of sRNAs in barley is locus-, chromosome- and/or cultivar-dependent and that a close relationship exists between sRNA size and sRNA type. These provide a fundamental understanding on how sRNAs are generated and expressed and function in plants. The discovery of differences in sRNA expression profile between different barley cultivars would provide a further understanding of the genetic structure and control of sRNA expression.

## Additional files

Additional file 1: Supplemental Methods. (PDF 295 kb) Additional file 2: Figure S1. Growth curve of shoot area of GP and Pallas under well watered and drought treatments. The projected shoot area is the sum of pixels from the two side view images and top view image taken (projected shoot area = side view 1 + side view 2 + top view). **Figure S2**. Size distribution of unique reads and read counts for GP and Pallas. A. Size distribution comparison of read counts between GP and Pallas (left) and Relative read frequency (Percentage) as a function of read length for GP and Pallas (right); B. Size distribution comparison of unique reads between GP and Pallas (left) and Relative frequency of unique reads (Percentage) for GP and Pallas (right); C. Size distribution of the genome-mapped read counts between GP and Pallas (left) and Relative frequency (Percentage) of genome-mapped read counts between GP and Pallas as a function of read length (right); D. Size distribution of the genome-mapped unique reads between GP and Pallas as a function of read length (left) and Relative frequency of genome mapped unique reads between GP and Pallas as a function of read length (right). **Figure S3**. The percentage of RNA types for GP and Pallas. A. The percentage of RNA types using the genome-mapped read count between GP and Pallas; B. The percentage of each mapped element by the genome-mapped unique reads between GP and Pallas; C. The logarithm of the percentage of each mapped element in Pallas to the percentage of each mapped element in GP. **Figure S4**. The percentage of all types of sRNAs in each size of the genome-mapped reads in Pallas and in GP. **Figure S5**. Quantitative real-time PCR of miRNAs in leaf tissues of GP and Pallas under water and drought conditions. Total RNA samples were polyadenlyated and reverse-transcribed using the NCode™ VILO™ miRNA cDNA Synthesis Kit (Invitrogen, Carlsbad, CA). Generated cDNAs were amplified with a miRNA-specific forward primer and the RT primer under the following condition: 3 min at 95 °C followed by 45 cycles of 1 s at 95 °C, 1 s at 55 °C, 30 s at 72 °C (fluorescence reading acquired) and 15 s at 81 °C. Normalization was performed using three biological replicates and four control genes as previously described [42]. **Figure S6**. Overrepresented functional categories for genes targeted by GP upregulated miRNAs (A) and Pallas up-regulated miRNAs (B). For each graphic, we extracted all functional GOslim terms that were 1.5 more frequent in the target genes compared to the background (all barley genes). The relative percentage of genes with a given GOslim term is depicted. For example, around 45 % of all GP target genes (in overrepresented functional categories) are involved in ion binding while this category is absent in Pallas. Ion binding and response to stresses are the only two functional categories that are statistically significantly more frequent among GP target genes than expected by chance alone. (PDF 385 kb) Additional file 3: Table S1. Sequencing and mappings statistics (Morex barley) for Reads from GP and Pallas reads. (DOC 26 kb) Additional file 4: Table S2. Cross comparison of reads from GP and Pallas. (XLSX 1351 kb) Additional file 5: Table S3. Cultivar-specific and differentially expressed sRNAs in GP and Pallas. (DOC 26 kb) Additional file 6: Table S4. sRNA types in GP and Pallas. (XLSX 16 kb) Additional file 7: Table S5. Percentage of mapped reads to the different chromosomes as a function of read length. (XLSX 32 kb) Additional file 8: Table S6. Individual genomic regions (contigs) mapped by sRNAs from GP and Pallas (Percentage of mapped reads). (XLSX 2587 kb) Additional file 9: Table S7. miRNAs detected in GP and Pallas. (XLSX 25 kb) Additional file 10: Table S8. Targets of up-regulated miRNAs in GP. (XLSX 48 kb) Additional file 11: Table S9. Targets of up-regulated miRNAs in Pallas. (XLSX 16 kb) Additional file 12: Table S10. Functional analysis of microRNA target genes using GO annotation (GO slim). (XLSX 44 kb)

## Abbreviations

AGO: Argonaute
BAC: bacterial artificial chromosome
CDS: coding sequence
GO: Gene Ontology
GP: Golden Promise
DCL: Dicer-like
LTR: long terminal repeat
miRNA: microRNA
nat-siRNA: natural antisense transcript siRNA
nt: nucleotide
phasiRNA: phased siRNA
pre-miRNA: hairpin precursor
pri-miRNA: miRNA primary transcript
qRT-PCR: quantitative real-time PCR
RDR: RNA-dependent RNA polymerase
RISC: RNA-induced silencing complex
RPM: reads per million
rRNA: ribosomal RNA
siRNA: short interfering RNA
snoRNA: small nucleolar RNA
snRNA: small nuclear RNA
sRNA: small RNA
ta-siRNA: trans-acting siRNA
TE: transposable element
tRNA: transfer RNA
UTR: untranslated region
WGS: whole genome shotgun
