# Supplementary material for: Generation of different sizes and classes of small RNAs in barley is locus, chromosome and/or cultivar-dependent
Source: BMC Genomics. 2016 Sep 15;17:735. doi: 10.1186/s12864-016-3023-5 (PMC5025612; doi:10.1186/s12864-016-3023-5)
Supplement: Additional file 1: — Supplemental Methods. (PDF 295 kb) [file 12864_2016_3023_MOESM1_ESM.pdf]

Supplemental Methods

1. Bioinformatics analysis

Bioinformatics analysis was performed using sRNAbench (Barturen et al., 2014), a new tool based on miRanalyzer (Hackenberg et al., 2011). Briefly, we first detected and removed the adapter, collapse reads into unique reads (non-redundant reads) and removed reads that contained ambiguous bases. The adapter cleaned reads were then mapped by means of the Bowtie (Langmead et al., 2009) to the Barley genome 030312v2 patch 22 (based on Morex downloaded from http://plants.ensembl.org/Hordeum_vulgare/Info/Index), Bowman and Barke genome assemblies allowing 0 mismatches. The genome mapped reads were then assigned to the different RNA types using the genome annotations and following databases:

1. miRBase microRNAs from release 20 (June 2013);
2. EnsemblPlants annotation in gtf format for 030312v2.22 downloaded from http://plants.ensembl.org/info/website/ftp/index.html. This annotation includes tRNA, snRNA and snoRNA;
3. Genomic tRNA database (http://gtrnadb.ucsc.edu/);
4. Ribosomal rRNA fragments extracted from Rfam;
5. Several repeat related databases: TREP, TIGR (Hordeum and Oryza Repeats, v3.0 and v3.3 respectively);
6. Chloroplast annotation extracted from the NC_008590 (http://www.ncbi.nlm.nih.gov/nuccore/NC_008590).

For each RNA element we calculated the total number of assigned reads and the normalized read count using RPM (Reads Per Million) calculated as 10^6 times the fraction between the number of reads mapped to a given RNA (or RNA type) and the total number of genome mapped reads.

2. Prediction and Gene Ontology (GO) analysis of miRNAs' targets

In order to detect differentially expressed miRNAs, we first applied a previously published method to detect novel miRNAs in both Pallas and GP (Hackenberg ET AL.,

2012). Those novel miRNAs were then pooled together with the known miRNAs from miRBase. Differentially expressed miRNAs were defined those with a higher than 2 fold-change between the two cultivars. The differentially expressed miRNAs were divided into two sets, those expressed stronger in GP and those expressed at higher levels in Pallas. For both sets independently, we predicted the target genes applying psRNATarget (Dai and Zhao, 2011) on the barley genes downloaded from ftp://ftp.ensemblgenomes.org/pub/release-22/plants/fasta/hordeum_vulgare/cdna/.

Next, we used Biomart from EnsemblPlants (http://plants.ensembl.org/biomart/martview/859a367ee9f990d20831ca48abf8f9b9) to obtain the GOslim terms (The GO Consortium, 2008) for barley. By means of the GOslim functional annotation we performed a functional *in silico* analysis based on AnnotationModules (Hackenberg and Matthiesen, 2008). Briefly, for each of the functional GOslim terms, the following steps were adopted:

1. Determine the total number of barely genes that have assigned the given GOslim term;
2. Determine the number of target genes that have assigned the given GOslim term;
3. Calculate the relative enrichment: R_e = (fraction of target genes with GOslim annotation) / (fraction of barley genes with GOslim annotation). If Re > 1, the GOslim term is more frequent among target genes than in the background (all barley genes) and Re < 1, means that the GOslim term is depleted among target genes;
4. Calculate a p-value for the null hypothesis Re=1 by means of the Fisher exact test;
5. Apply Benjamini-Hochberg (Benjamini et al., 1995) correction for multiple testing.

3. Genome distribution

The genome distribution plots (Figure 5A and B) are based on the Bowtie alignment files. Each chromosome is divided into 1000 intervals. For each interval, we determine the number of mapped unique reads and the total read count as a function of read length. The start coordinate determines if a read is assigned to a given interval and we

don't distinguish between the two strands. Finally, we normalize the read counts for each read length, i.e. the sum of all interval counts over the different chromosomes and scaffolds yields 100 for each read length. Therefore, the peak heights cannot be compared between the different read lengths – only the chromosome distribution for a given read length can be analysed.
