# Supplementary material for: Generation of different sizes and classes of small RNAs in barley is locus, chromosome and/or cultivar-dependent
Source: BMC Genomics. 2016 Sep 15;17:735. doi: 10.1186/s12864-016-3023-5 (PMC5025612; doi:10.1186/s12864-016-3023-5)
Supplement: Additional file 2: Figure S1. — Growth curve of shoot area of GP and Pallas under well watered and drought treatments. The projected shoot area is the sum of pixels from the two side view images and top view image taken (projected shoot area = side view 1 + side view 2 + top view). Figure S2. Size distribution of unique reads and read counts for GP and Pallas. A. Size distribution comparison of read counts between GP and Pallas (left) and Relative read frequency (Percentage) as a function of read length for GP and Pallas (right); B. Size distribution comparison of unique reads between GP and Pallas (left) and Relative frequency of unique reads (Percentage) for GP and Pallas (right); C. Size distribution of the genome-mapped read counts between GP and Pallas (left) and Relative frequency (Percentage) of genome-mapped read counts between GP and Pallas as a function of read length (right); D. Size distribution of the genome-mapped unique reads between GP and Pallas as a function of read length (left) and Relative frequency of genome mapped unique reads between GP and Pallas as a function of read length (right). Figure S3. The percentage of RNA types for GP and Pallas. A. The percentage of RNA types using the genome-mapped read count between GP and Pallas; B. The percentage of each mapped element by the genome-mapped unique reads between GP and Pallas; C. The logarithm of the percentage of each mapped element in Pallas to the percentage of each mapped element in GP. Figure S4. The percentage of all types of sRNAs in each size of the genome-mapped reads in Pallas and in GP. Figure S5. Quantitative real-time PCR of miRNAs in leaf tissues of GP and Pallas under water and drought conditions. Total RNA samples were polyadenlyated and reverse-transcribed using the NCode™ VILO™ miRNA cDNA Synthesis Kit (Invitrogen, Carlsbad, CA). Generated cDNAs were amplified with a miRNA-specific forward primer and the RT primer under the following condition: 3 min at 95 °C followed by 45 cycles of 1 s at 95 °C, 1 s at 55 [file 12864_2016_3023_MOESM2_ESM.pdf]

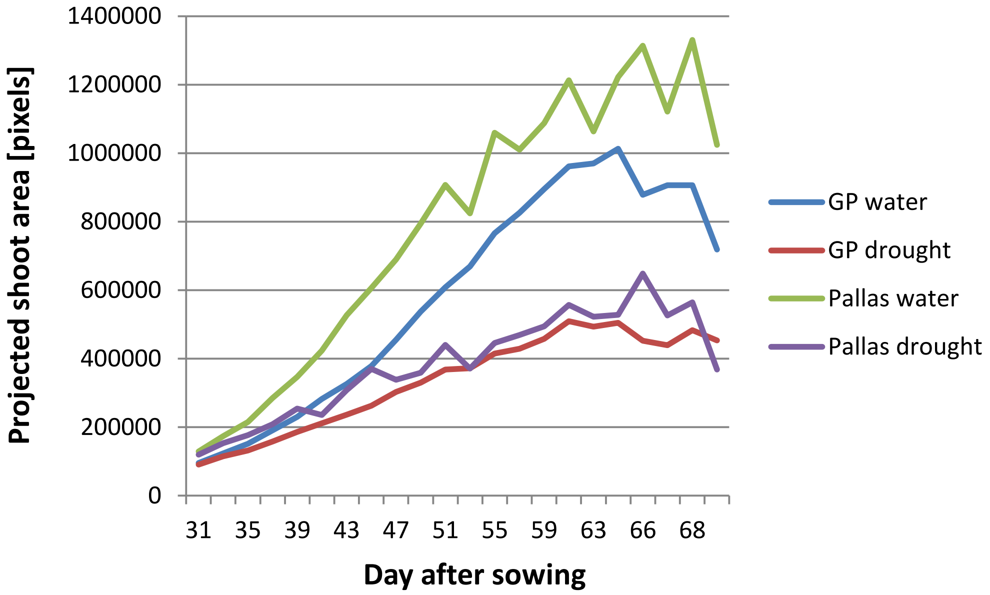

Supplemental Figure 1

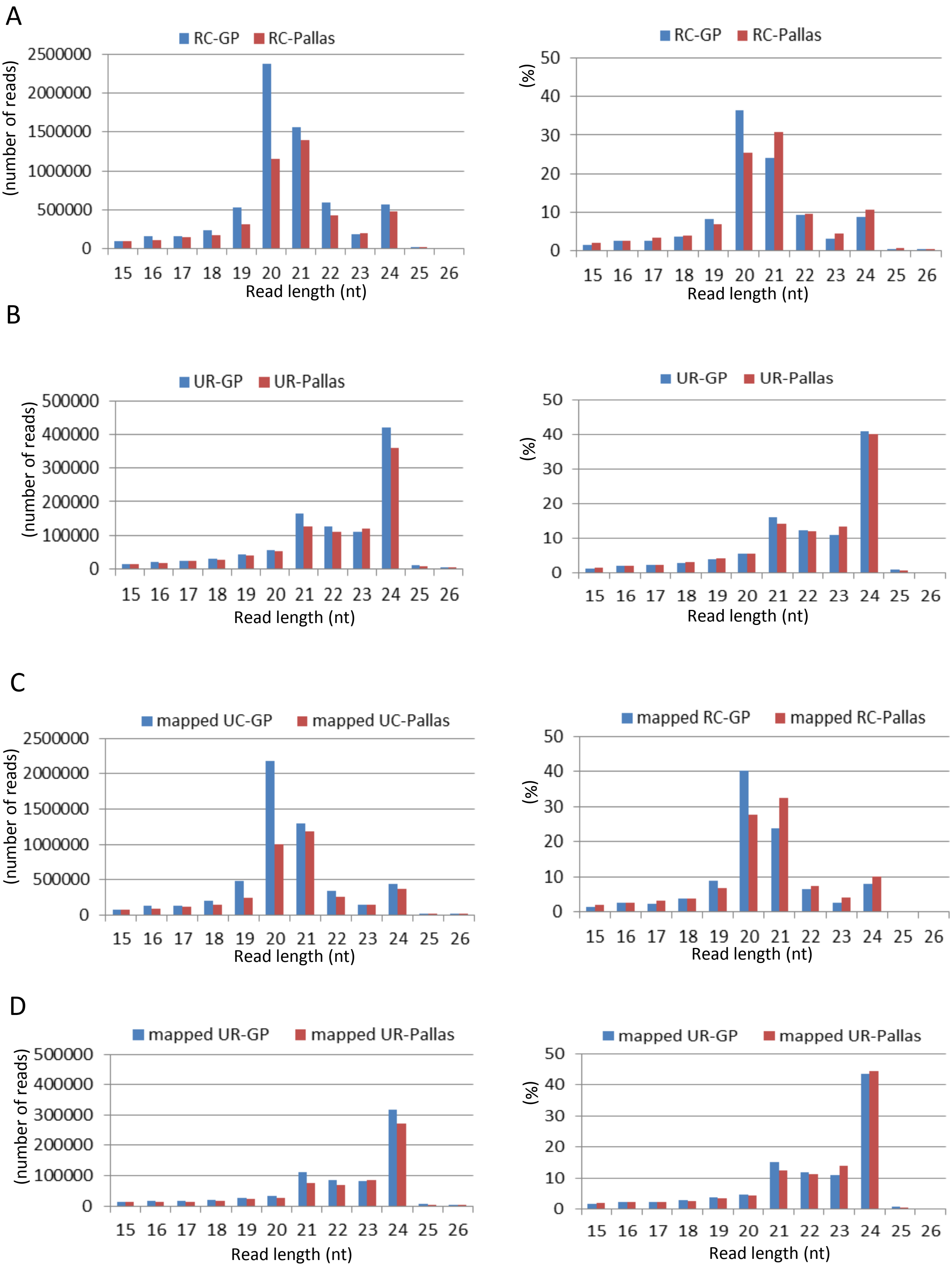

Supplemental Figure 2

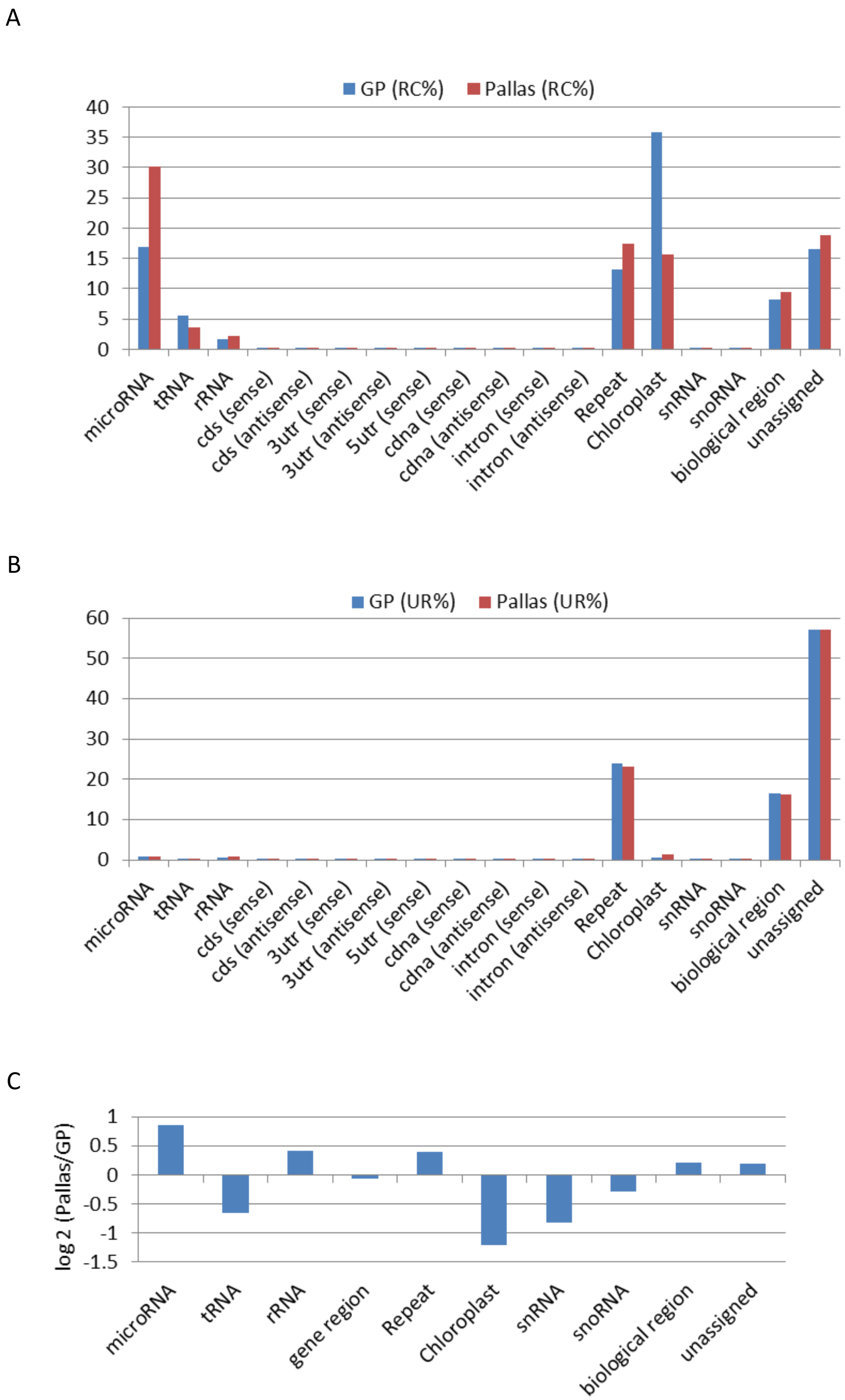

Supplemental Figure 3

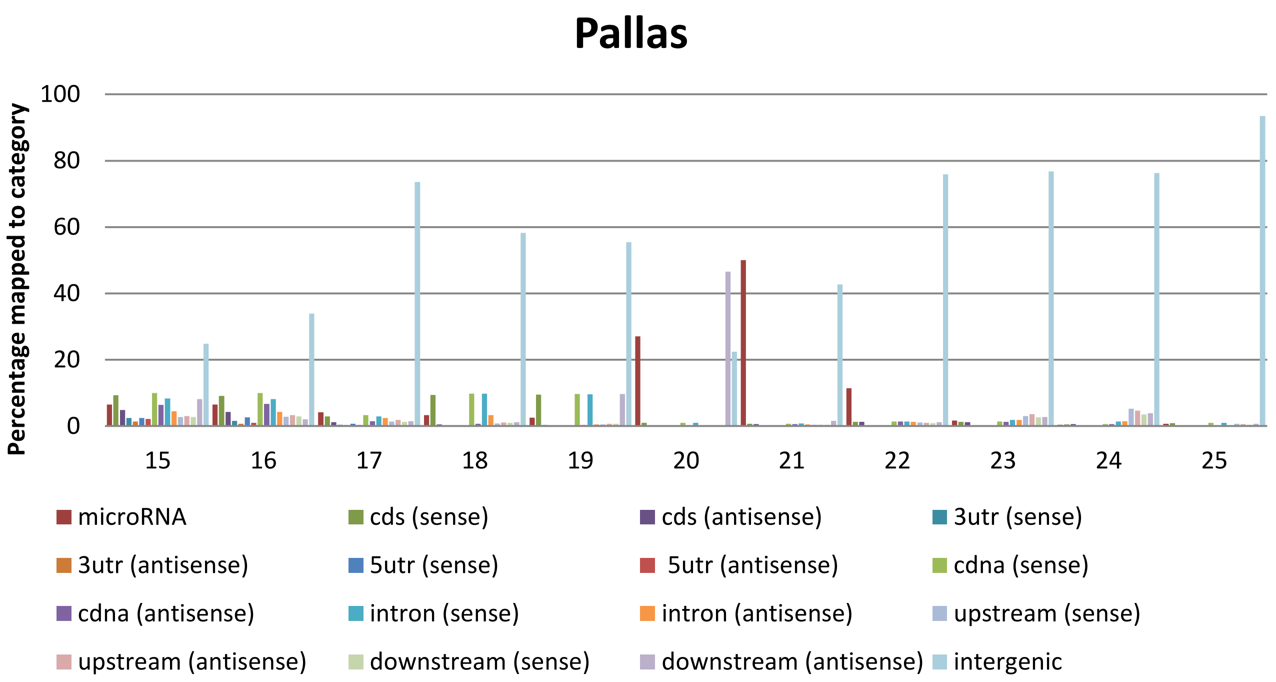

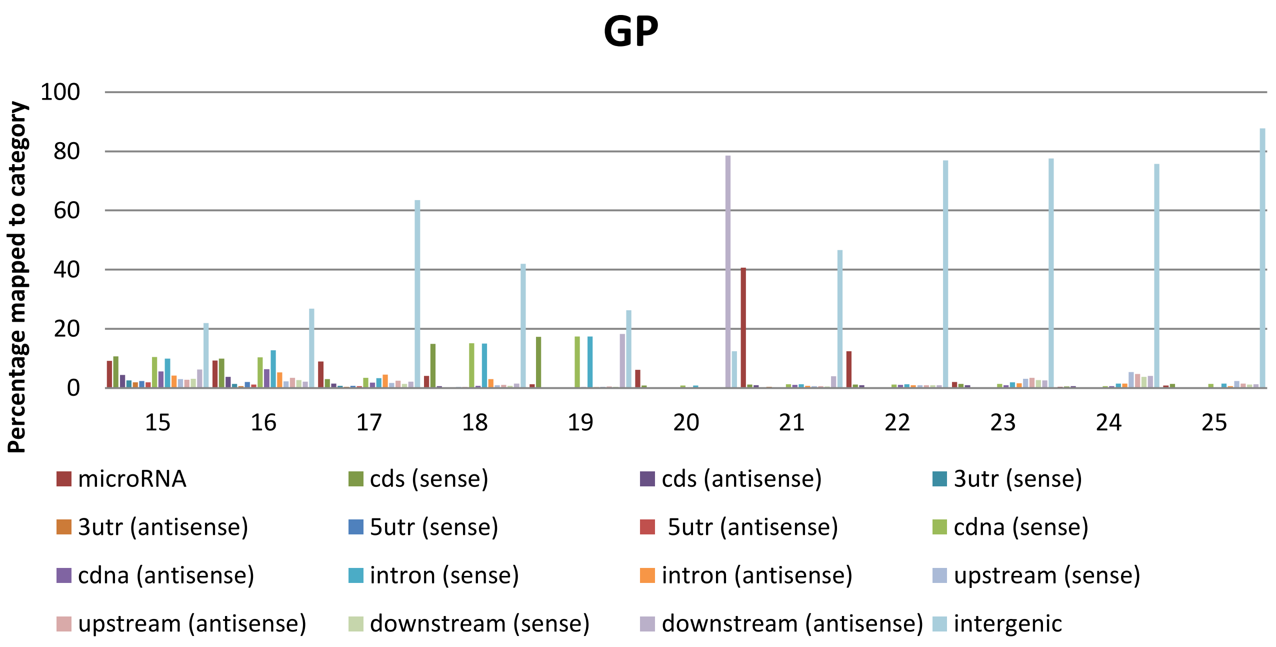

Supplemental Figure 4

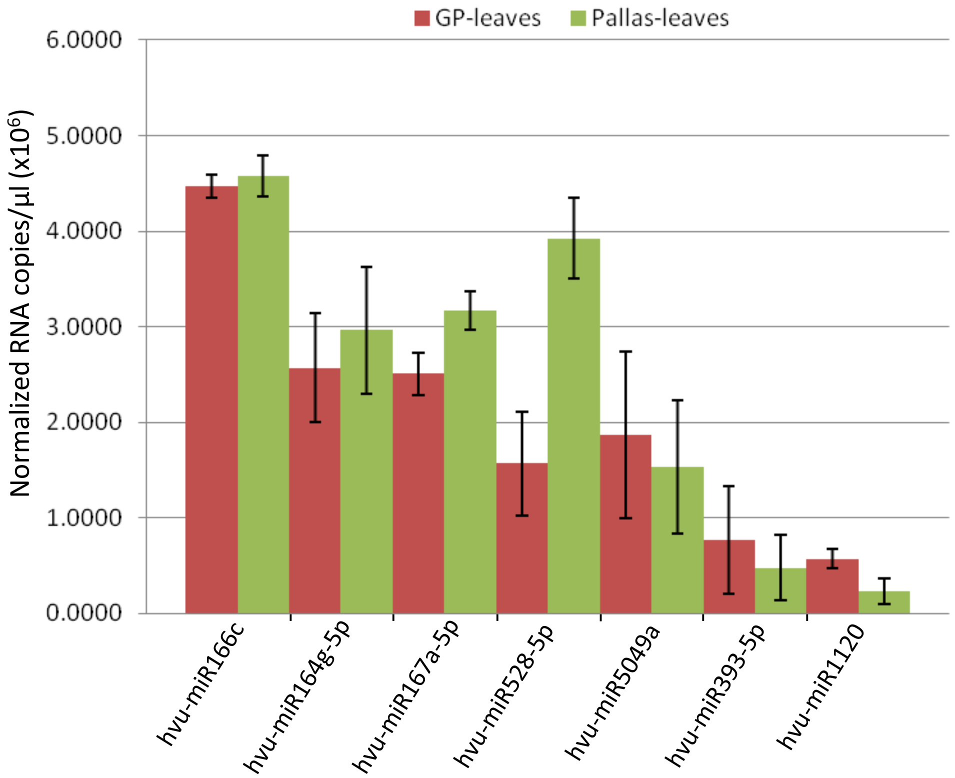

Supplemental Figure 5

A

GP

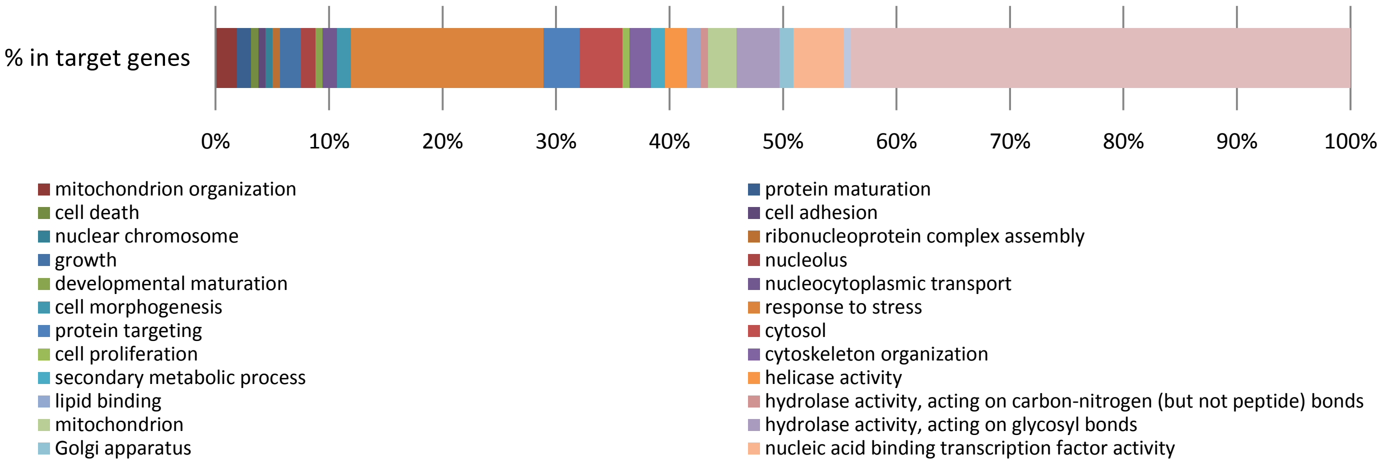

B

Pallas

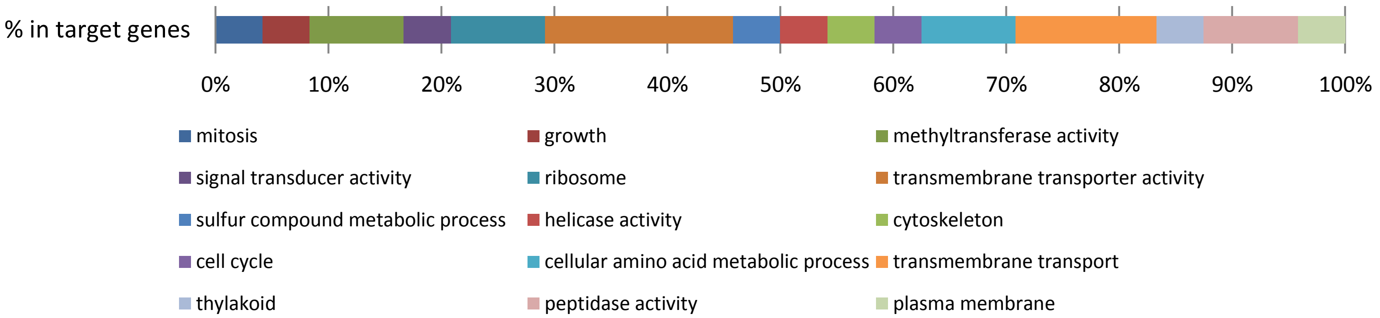

Supplemental Figure 6
