# Supplementary material for: Generation of different sizes and classes of small RNAs in barley is locus, chromosome and/or cultivar-dependent
Source: BMC Genomics. 2016 Sep 15;17:735. doi: 10.1186/s12864-016-3023-5 (PMC5025612; doi:10.1186/s12864-016-3023-5)
Supplement: Additional file 3: Table S1. — Sequencing and mappings statistics (Morex barley) for Reads from GP and Pallas reads. (DOC 26 kb) [file 12864_2016_3023_MOESM3_ESM.doc]

Supplemental Table 1. Reads from GP and Pallas mapped to the genome of Morex barley

| **cultivar** | **raw read** | **clean read count (RC)*** | **clean unique read (UR)*** | **genome-mapped RC** | **genome-mapped UR** | **% genome-mapped RC** | **% genome-mapped UR** |
| --- | --- | --- | --- | --- | --- | --- | --- |
| Pallas | 5370783 | 4509869 | 898728 | 3626535 | 612278 | 80.41 | 68.12 |
| GP | 7113852 | 6482301 | 1023846 | 5424790 | 725545 | 83.69 | 70.86 |

“*”: after the removal of adapter and reads of <15 nt or >26 nt and quality filter.
