# Supplementary material for: Generation of different sizes and classes of small RNAs in barley is locus, chromosome and/or cultivar-dependent
Source: BMC Genomics. 2016 Sep 15;17:735. doi: 10.1186/s12864-016-3023-5 (PMC5025612; doi:10.1186/s12864-016-3023-5)
Supplement: Additional file 5: Table S3. — Cultivar-specific and differentially expressed sRNAs in GP and Pallas. (DOC 26 kb) [file 12864_2016_3023_MOESM5_ESM.doc]

Supplemental Table 2. Cross comparison of reads from GP and Pallas

| **cultivar** | **non-shared unique reads (UR)** | **non-shared read count (RC)** | **shared UR** | **shared RC** | **% shared RC** | **% shared UR** |
| --- | --- | --- | --- | --- | --- | --- |
| GP | 618839 | 748936 | 106706 | 4675854 | 86.19 | 14.71 |
| Pallas | 505572 | 613781 | 106706 | 3012754 | 83.08 | 17.43 |
